# Successful treatment of coexisting membranous nephropathy and immune thrombocytopenia by eradicating gastric *Helicobacter pylori* infection: a case report

**DOI:** 10.1007/s13730-023-00805-7

**Published:** 2023-07-08

**Authors:** Reina Suetsugu, Hirofumi Sakuma, Keisuke Maruyama, Motoki Matsuki, Yayoi Ogawa, Naoki Nakagawa

**Affiliations:** 1 Division of Cardiology, Nephrology, Pulmonology, and Neurology, Department of Internal Medicine, Asahikawa Medical University, Midorigaoka-Higashi 2-1-1-1, Asahikawa, Japan; 2 Hokkaido Renal Pathology Center, Sapporo, Japan

**Keywords:** Helicobacter, Membranous nephropathy, Nephrotic syndrome, Thrombocytopenia

## Abstract

Membranous nephropathy (MN) is a common cause of nephrotic syndrome in middle-aged and older adults. MN etiology is mainly primary or idiopathic; however, it may also be secondary to infections, drugs, neoplasms, and autoimmune diseases. We present the case of a 52-year-old Japanese man with coexisting nephrotic MN and immune thrombocytopenic purpura (ITP). Renal biopsy revealed glomerular basement membrane thickening with immunoglobulin (Ig) G and complement component 3 deposition. Glomerular IgG subclass analysis revealed predominant IgG4 deposition with weak IgG1 and IgG2 deposition. IgG3 and phospholipase A2 receptor deposits were negative. Upper endoscopy revealed no ulcers, but histological examination demonstrated *Helicobacter pylori* infection in the gastric mucosa with elevated IgG antibodies. After gastric *Helicobacter pylori* eradication, the nephrotic-range proteinuria and thrombocytopenia of the patient were markedly improved without initiation of immunosuppressive treatment. Therefore, clinicians should consider the possibility of *Helicobacter pylori* infection in patients with coexisting MN and ITP. Further studies are required to demonstrate the associated pathophysiological aspects.

## Introduction

Membranous nephropathy (MN) is a common cause of nephrotic syndrome in middle-aged and older patients [1, 2]. In most cases, its etiology is idiopathic; however, it may occur secondary to several causes, including infections, drugs, neoplasms, and autoimmune diseases [3–5]. Recently, reports have indicated a pathogenic correlation between *Helicobacter pylori* infection and MN [6–8]. *Helicobacter pylori* antigens have been found in the glomeruli of MN patients [9], and successful *Helicobacter pylori* eradication reduced proteinuria [6, 7]. Additionally, immune thrombocytopenic purpura (ITP), an autoimmune bleeding disorder characterized by platelet destruction and production inhibition [10], has a pathophysiological link with chronic *Helicobacter pylori* infections. This finding is based on the increased platelet count of patients with ITP after gastric *Helicobacter pylori* eradication therapy [11, 12].

Herein, we present the case of a 52-year-old Japanese man with coexisting MN and ITP whose nephrotic-range proteinuria and thrombopenia were markedly improved after the eradication of gastric *Helicobacter pylori* infection.

## Case report

A 52-year-old Japanese man with a history of hypertension and dyslipidemia was admitted to our hospital. His annual health check-ups revealed no significant blood or urine test abnormalities besides dyslipidemia. However, 3 months before the admission, he developed edema in his lower limbs and high dipstick proteinuria (3+).

On evaluation, the patient was 168 cm tall and weighed 87 kg, which increased by 3 kg compared to before the edema appeared. He had a 128/84 mmHg blood pressure and an 84 bpm pulse with a regular rhythm. Lower limb edema with pinpoint-sized petechiae was evident. Neurological findings were unremarkable. He had no symptoms or findings suggesting autoimmune diseases, including systemic lupus erythematosus or Sjögren's syndrome. Table 1 demonstrates the laboratory findings on admission. His spot urinary protein-to-creatinine ratio was 7.6 g/g Cr with a decreased serum albumin concentration of 2.1 g/dL, leading to the diagnosis of nephrotic syndrome. Notably, his serum creatinine concentration was 0.60 mg/dL. His platelet count dropped to 54,000 /μL, below the ITP diagnostic criterion of 100,000 /μL.

**Table 1:** Laboratory findings on admission

Urinalysis		Chemistry		Serology (normal range)	
Specific gravity	1.022	Total protein	5.5 g/dL	Rheumatoid factor	< 3.0 IU/mL (< 15 IU/mL)
pH	6.5	Albumin	2.0 g/dL	IgG	1136.8 mg/dL
Protein	(3+)	AST	21 IU/L	IgA	339.1 mg/dL
Glucose	(–)	ALT	16 IU/L	IgM	56.6 mg/dL
Occult blood	(2+)	LDH	188 IU/L	C3	123.4 mg/dL
Sediment		Total cholesterol	279 mg/dL	C4	33.2 mg/dL
RBC	20–29/hpf	Triglyceride	112 mg/dL	CH50	47.5 U/mL
WBC	1–4/hpf	BUN	12.7 mg/dL	ANA	40× (< 40×)
U-PCR	7.6 g/gCr	Creatinine	0.61 mg/dL	dsDNA	0.5 IU/mL (< 10 IU/mL)
U-NAG/Cr	14.3 U/gCr	eGFR	109 mL/min/1.73m^2^	ssDNA	12 AU/mL (< 25 AU/mL)
U-β2MG	0.20 μg/mL	Cys C	0.70 mg/L	RNP	1.1 U/mL (< 5.0 U/mL)
Ccr	115.1 mL/min	Na	140 mEq/L	Sm	33.0 U/mL (< 7.0 U/mL)
		K	4.0 mEq/L	SS-A	> 240.0 U/mL (< 7.0 U/mL)
Peripheral blood		Cl	107 mEq/L	SS-B	0.7 U/mL (< 7.0 U/mL)
White blood cell	7120/μL	Ca	8.3 mg/dL	PA-IgG	53.0 ng/10^7^cells (< 46 ng/10^7^cells)
Red blood cell	385 × 10^4^/μL	iP	2.7 mg/dL	Cryoglobulin	–
Hemoglobin	11.6 g/dL			HBs-Ag	–
Hematocrit	34.6%			HCV-Ab	–
Platelet	54 × 10^4^/μL			C-reactive protein	0.88 mg/dl

*hpf* high power field, *β2MG* β2-microglobulin, *Ccr* creatinine clearance, *AST* aspartate aminotransferase, *ALT* alanine aminotransferase, *LDH* lactate dehydrogenase, *ALP* alkaline phosphatase, *BUN* blood urea nitrogen, *ANA* anti-nuclear antibody, *MPO-ANCA* myeloperoxidase-antineutrophil cytoplasmic antibody, *PR3-ANCA* Proteinase 3-antineutrophil cytoplasmic antibody, *anti-GBM* anti-glomerular basement membrane, *ACE* angiotensin-converting enzyme

**Fig. 1:**
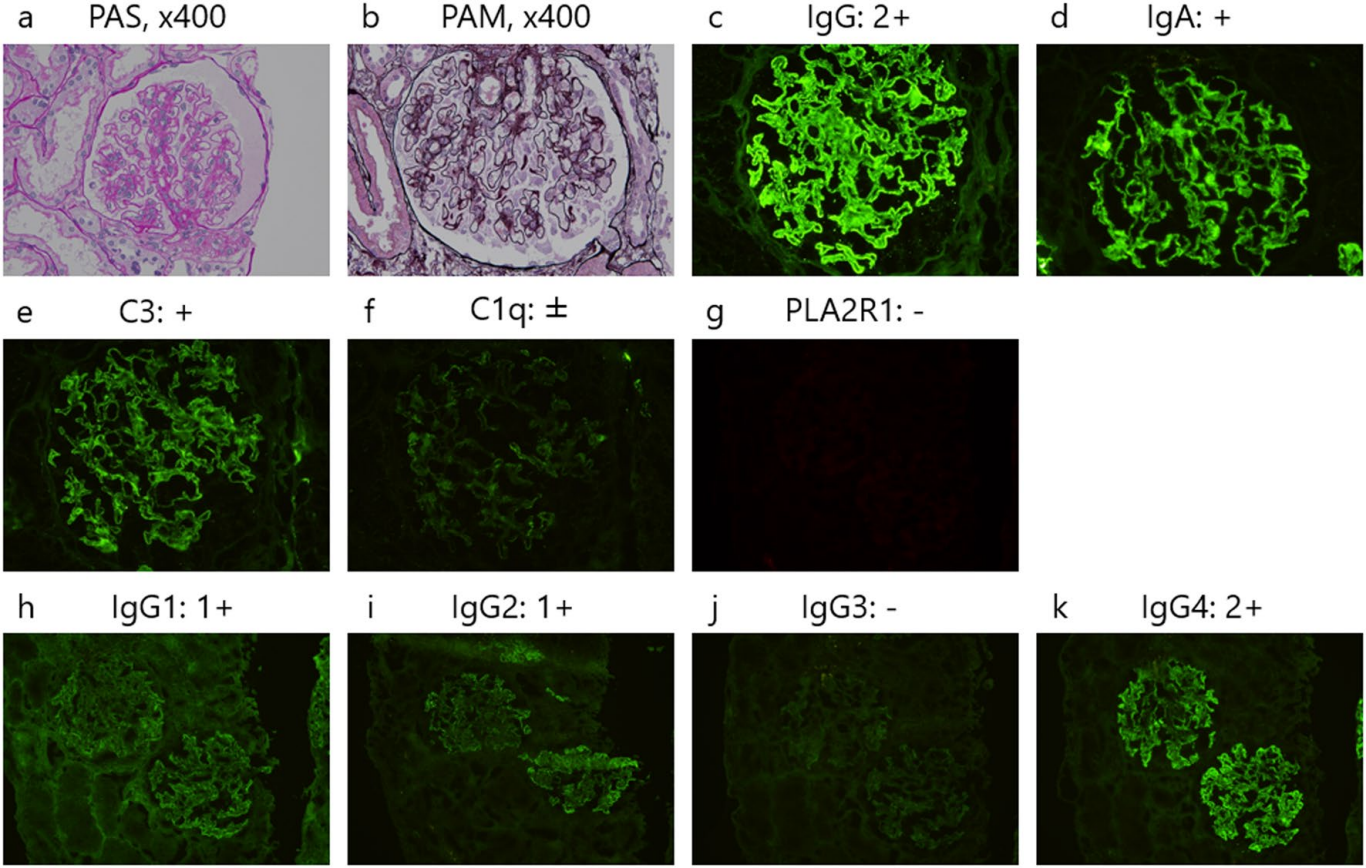
Light microscopy and immunofluorescence findings of the renal biopsy. **a** Light micrograph reveals 25 glomeruli without global sclerosis (periodic acid–Schiff (PAS) staining, ×400). No segmental sclerotic lesions can be observed. **b** The glomerular basement membrane was thickened with a bubbly appearance and a spike lesion (periodic acid–methenamine silver (PAM) staining, ×400). **c–f** Immunofluorescence staining reveals deposition of IgG (**c**), IgA (**d**), and C3 (**e**) and trace staining for C1q (f) in the glomerular basement membrane (×400). **g** Staining for PLA2R is negative (×400). **h–k** Immunofluorescence analysis of IgG subclasses indicates a predominance of IgG4 deposition (**k**) followed by IgG1 (**h**) and IgG2 (**i**) in a peripheral granular pattern (×200). The staining for IgG3 is negative (**j**). *Ig* immunoglobulin, *C3* complement component 3, *PLA2R* phospholipase A2 receptor

**Fig. 2:**
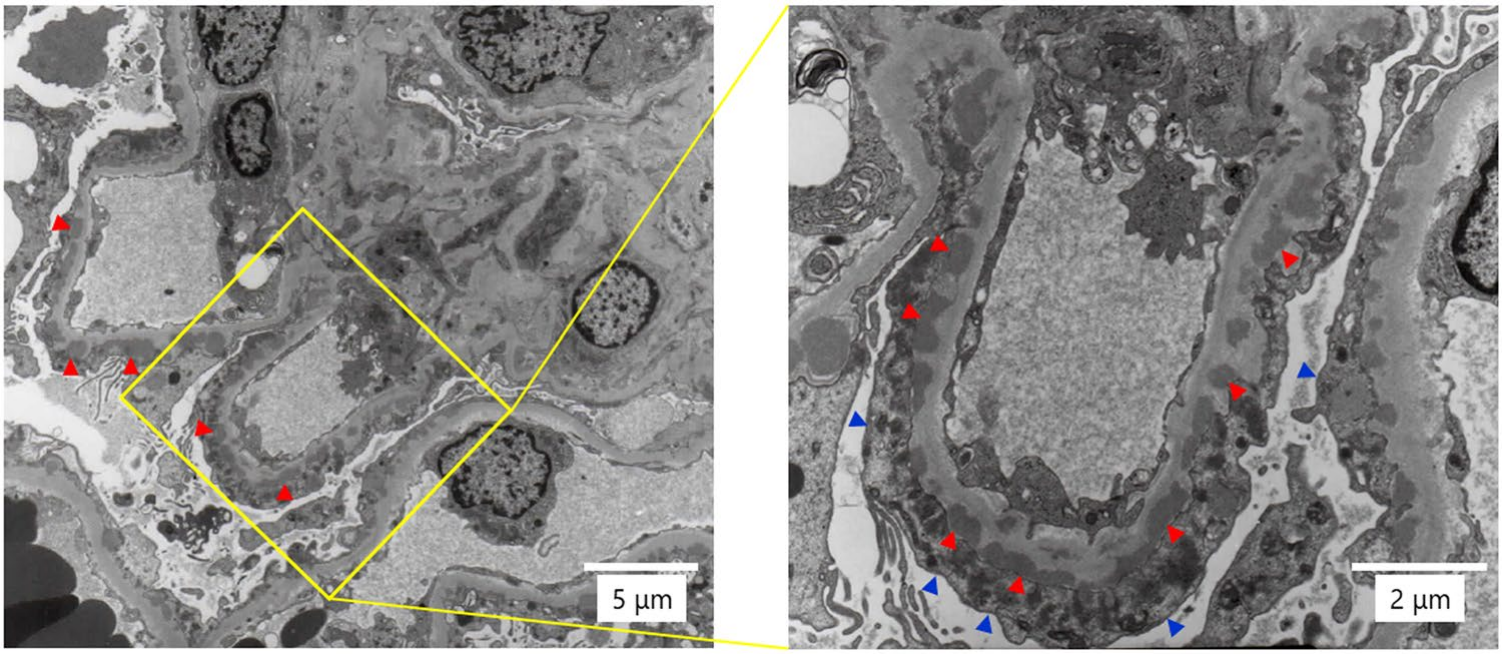
Transmission electron microscopic findings of the renal biopsy. The transmission electron micrograph of the renal tissue reveals granular electron-dense deposits in the subepithelial regions of the glomerular basement membrane (red arrowheads). Diffuse podocyte effacement can be observed (blue arrows)

Figure 1 illustrates the renal biopsy findings performed 1 day after admission. Light microscopy revealed 25 glomeruli without global glomerulosclerosis (Fig. 1a). The glomerular basement membrane (GBM) was thickened with a bubbly appearance and spike lesion. However, mesangial proliferation, endocapillary hypercellularity, extra-capillary proliferation, or segmental sclerotic lesions were not observed. Immunofluorescence staining revealed that the capillary walls were strongly positive for immunoglobulin (Ig) G and mild for IgA (Fig. 1d), complement component 3 (C3) (Fig. 1e), trace staining for IgM andC1q (Fig. 1f), and negative for phospholipase A2 receptor (PLA2R) (Fig. 1g). Glomerular IgG subclass analysis revealed predominant IgG4 deposition (Fig. 1k) but weak IgG1 and IgG2 deposition (Fig. 1h, i). The IgG3 deposit was negative (Fig. 1j). Transmission electron microscopy revealed granular electron-dense deposits in the subepithelial regions of the GBM (Fig. 2). Additionally, signs of podocyte damage were observed, including diffuse foot process effacement and an increase in the number of actin filaments. These histological features were compatible with the diagnosis of secondary MN (Ehrenreich and Churg criteria stage II). Malignancy was almost ruled out using upper and lower gastrointestinal endoscopy and whole-body computed tomography scans. Upper endoscopy revealed no ulcers, but histological examination demonstrated *Helicobacter pylori* infection in the gastric mucosa. Table 1 reveals the negative test results for serum anti-hepatitis B surface antigen and anti-hepatitis C virus antibodies. The autoimmune screen revealed positive anti-nuclear antibodies, anti-Smith (Sm) antibodies (33.0 U/mL; normal range < 7.0 U/mL), and anti-Sjögren's-syndrome-related antigen A (SS-A) antibodies (> 240 U/mL; normal range < 7.0 U/mL), but was negative for double-stranded deoxyribonucleic acid and ribonucleic acid-polymerase-III antibodies. Complement levels and thyroid function tests were within their normal ranges without any subjective or objective findings associated with autoimmune diseases.

One week after the renal biopsy, the patient's platelet-associated-IgG and *Helicobacter pylori* IgG antibodies of the patient were elevated at 53 ng/10^7^ cells (> 46 ng/10^7^ cells) and 30.9 U/mL (> 9.9 U/ml), respectively. A bone marrow aspirate demonstrated normal cellularity, adequate megakaryocytes, and no evidence of malignancy. There were no other apparent thrombocytopenia causes including medications and food supplements. However, his 3-month platelet count of 67,000–88,000 /μL indicated the presence of ITP. From these results, we speculated that *Helicobacter pylori* infection caused secondary MN and ITP. The patient was started on triple therapy for his *Helicobacter pylori* infection (amoxicillin 1 g twice daily, clarithromycin 500 mg twice daily, and pantoprazole 40 mg twice daily for 14 days).

**Fig. 3:**
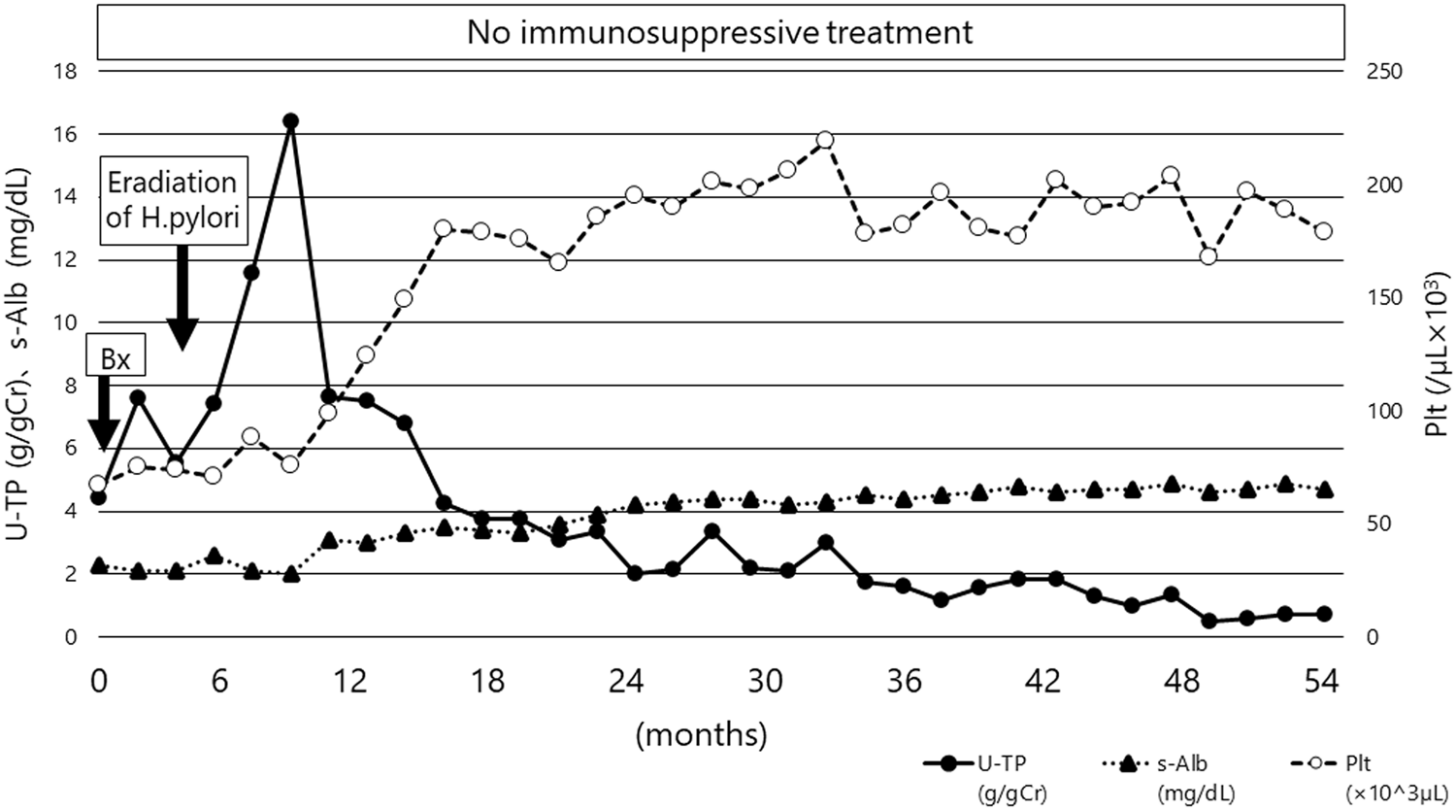
Clinical course after admission to our hospital. Gastric *Helicobacter pylori* eradiation causes a proteinuria decrease and a gradual platelet count increase. *U-pro* urinary protein, *g/gCr* urinary protein/creatinine ratio

Four months after the *Helicobacter pylori* infection was eradicated, the proteinuria and thrombocytopenia of the patient improved without initiation of immunosuppressive treatment (Fig. 3). Furthermore, his serum albumin concentration increased to 4.0 g/dL, and proteinuria decreased within the normal range, indicating complete remission. Additionally, his platelet-associated IgG titer decreased within the normal range (34 ng/10^7^ cells) after 4 months post-eradication. After 5 years post-eradication, although anti-nuclear, anti-Sm, and anti-SS-A antibodies were still positive (40×, 13.0 U/mL, and > 240 U/mL, respectively), the patient did not exhibit any symptoms of autoimmune disease or MN and ITP relapse. Therefore, we hypothesized that *Helicobacter pylori* infection was associated with the coexistence of MN and ITP.

## Discussion

This study is the first case report to describe the successful treatment of coexisting MN and ITP solely through eradicating gastric *Helicobacter pylori* infection.

MN is the most common cause of nephrotic syndrome among adults, accounting for approximately one-third of cases. It is especially prevalent in Caucasian adults, with a 2:1 male preponderance and a peak incidence in individuals aged 50–60 years [5]. There is increasing evidence that autoimmune issues may cause idiopathic MN. Furthermore, this finding is supported by target antigens on the podocyte surface which have been found in most primary MN cases. These proteins are mainly PLA2R and rarely thrombospondin type 1 domain-containing 7A [5]. Serum PLA2R autoantibodies have been found in most (52–82%) patients with primary MN and are typically absent in those with secondary MN conditions [13]. Our patient tested negative for PLA2R in the immunofluorescence renal biopsy findings, indicating a possible secondary pathogenesis contributing to his MN. However, primary MN cannot be completely ruled out.

Furthermore, capillary wall positivity was present for IgG1, IgG2, as well as IgG4, and negative for IgG3. While autoimmune diseases, including systemic lupus erythematous, have long been associated with MN, other vague autoimmune syndromes have also been reported, including inflammatory arthritis and ITP [14–16]. Additionally, an association between *Helicobacter pylori* infection and autoimmune diseases has been previously reported, including SLE, Sjogren's disease, and rheumatoid arthritis [17]. Although our patient did not exhibit a typical full-house pattern of immunoglobulin deposition in the glomerular basement membrane or any symptoms suggesting autoimmune diseases, including systemic lupus erythematosus or Sjögren's syndrome, he may have had an underlying autoimmune disease or overlapping syndrome because he was positive for anti-Sm and anti-SS-A antibodies even after 5 years post-eradication of his gastric *Helicobacter pylori*. The anti-Sm antibody is specific for systemic lupus erythematosus; however, its sensitivity is restricted [18]. The clinical significance of anti-Sm antibodies is still under debate. Furthermore, although we must monitor our patient for the possibility of late-onset systemic lupus erythematosus [18] or overlap syndrome [19], we successfully treated coexisting MN and ITP with gastric *Helicobacter pylori* eradication.

Several cases have previously reported a link between MN as well as ITP and were treated using prednisolone [19], a combination of prednisolone with immunosuppressive therapy [15, 16], rituximab [20], or low-density lipoprotein apheresis [21]. However, there are no reports on patients with coexisting MN and ITP whose condition improved after gastric *Helicobacter pylori* infection eradication. A recent meta-analysis on the effect of eradicating gastric *Helicobacter pylori* in patients with ITP, including 241 patients (125 in the eradication group and 116 in the control group) from 6 studies, demonstrated that those in the eradication group had a significantly higher overall platelet response rate than those in the control group (odds ratio = 1.93, 95% confidence interval: 1.01–3.71, *P* = 0.05) [22]. This finding indicates that *Helicobacter pylori* eradication has a significant therapeutic effect on patients with ITP. Considering the intrinsic desing limitation and sample size of the included studies, physicians in the clinical scenario should consider eradicating of gastric *Helicobacter pylori* in patients with coexisting MN and ITP.

Our study had several limitations. First, we could not provide a direct relationship and time course evidence between MN, ITP, and *Helicobacter pylori* infection. Patient symptoms developed several months before admission; however, deposit localization in electron micrographs (Ehrenreich and Churg criteria stage II) and strong IgG staining suggested a long-lasting immune abnormality causing membranous nephropathy. Second, we cannot completely exclude the possibility that the symptoms of the patient could have improved by spontaneous MN and ITP remission. Spontaneous complete proteinuria remission in patients with MN occurs in 5 to 30 percent patient at 5 years [23–25]. Furthermore, spontaneous remissions (presumed to be treatment independent) occur in up to 10–20% adults with ITP [26, 27]. These often occur within the first 6 months, but platelet count improvements years later have also been reported [27]. Third, serum PLA2R and thrombospondin were not tested, which can be considered a limitation in our report. Furthermore, multiple studies have found autoantibodies bound to podocyte antigens, predominantly belonging to the IgG4 subclass. These podocyte-specific autoantibodies can be present in the serum and glomeruli of patients [11].

In conclusion, clinicians should consider the possibility of *Helicobacter pylori* infection in patients with coexisting MN and ITP. Further studies are required to demonstrate the associated pathophysiology.

## References

[1] Floege J, Amann K (2016). Primary glomerulonephritides. Lancet.

[2] Yokoyama H, Taguchi T, Sugiyama H, Sato H (2012). Membranous nephropathy in Japan: analysis of the Japan Renal Biopsy Registry (J-RBR). Clin Exp Nephrol.

[3] Yasuda I, Tokuyama H, Hashiguchi A, Hasegawa K, Uchiyama K, Ryuzaki M (2021). Malignancy-associated membranous nephropathy with PLA2R double-positive for glomeruli and carcinoma. CEN Case Rep.

[4] Kidney Disease: Improving Global Outcomes Glomerular Diseases Work G. KDIGO,  (2021). Clinical practice guideline for the management of glomerular diseases. Kidney Int.

[5] Ronco P, Debiec H (2021). Membranous nephropathy: current understanding of various causes in light of new target antigens. Curr Opin Nephrol Hypertens.

[6] Sugimoto T, Furukawa T, Maeda T, Somura M, Uzu T, Kashiwagi A (2007). Marked reduction of proteinuria after eradication of gastric Helicobacter pylori infection in a patient with membranous nephropathy: coincidental or associated?. Intern Med.

[7] Caliskan B, Yazici H, Caliskan Y, Ozluk Y, Gulluoglu M, Kilicaslan I (2014). The effects of *Helicobacter pylori* eradication on proteinuria in patients with primary glomerulonephritis. Int J Nephrol.

[8] Nesheiwat Z, Daboul J, Merugu GP, Adapa S, Balla M (2021). Membranous nephropathy and autoimmune hepatitis in the setting of acute Helicobacter pylori infection: a case report. J Med Case Rep.

[9] Nagashima R, Maeda K, Yuda F, Kudo K, Saitoh M, Takahashi T (1997). Helicobacter pylori antigen in the glomeruli of patients with membranous nephropathy. Virchows Arch.

[10] McMillan R (2007). The pathogenesis of chronic immune thrombocytopenic purpura. Semin Hematol.

[11] Frydman GH, Davis N, Beck PL, Fox JG (2015). *Helicobacter pylori* eradication in patients with immune thrombocytopenic purpura: a review and the role of biogeography. Helicobacter.

[12] Pellicano R, Ianiro G, Fagoonee S, Settanni CR, Gasbarrini A (2020). Review: extragastric diseases and *Helicobacter pylori*. Helicobacter.

[13] Dai H, Zhang H, He Y (2015). Diagnostic accuracy of PLA2R autoantibodies and glomerular staining for the differentiation of idiopathic and secondary membranous nephropathy: an updated meta-analysis. Sci Rep.

[14] Sawamura M, Sawa N, Yamanouchi M, Ikuma D, Sekine A, Mizuno H (2022). Use of biologic agents and methotrexate improves renal manifestation and outcome in patients with rheumatoid arthritis: a retrospective analysis. Clin Exp Nephrol.

[15] Kano K, Ito S, Ando T, Arisaka O, Tomita S, Ueda Y (1999). Chronic idiopathic thrombocytopenic purpura in a patient with membranous glomerulonephritis. Nephron.

[16] Tamura K, Takagi N, Yabana M, Kihara M, Toya Y, Takizawa T (1999). Nephrotic syndrome due to membranous glomerulonephritis in a patient with idiopathic thrombocytopenic purpura. Nephron.

[17] Etchegaray-Morales I, Jimenez-Herrera EA, Mendoza-Pinto C, Rojas-Villarraga A, Macias-Diaz S, Osorio-Pena AD (2021). *Helicobacter pylori* and its association with autoimmune diseases: systemic lupus erythematosus, rheumatoid arthritis and Sjogren syndrome. J Transl Autoimmun.

[18] van Beers J, Schreurs MWJ (2022). Anti-Sm antibodies in the classification criteria of systemic lupus erythematosus. J Transl Autoimmun.

[19] Lande MB, Thomas GA, Houghton DC (2001). Membranous nephropathy associated with chronic immune thrombocytopenic purpura in childhood. Am J Kidney Dis.

[20] Alkindi S, Khan S, Riyami D, Farooqi M, Pathare A (2010). Coexistence of immune thrombocytopenic purpura and idiopathic membranous glomerulonephritis successfully treated with rituximab. Platelets.

[21] Nishizawa K, Yamashita T, Ogawa Y, Kobayashi H (2022). Membranous nephropathy complicated by immune thrombocytopenia treated with low-density lipoprotein apheresis: a case report and literature review. CEN Case Rep.

[22] Kim BJ, Kim HS, Jang HJ, Kim JH (2018). *Helicobacter pylori* eradication in idiopathic thrombocytopenic purpura: a meta-analysis of randomized trials. Gastroenterol Res Pract.

[23] Schieppati A, Mosconi L, Perna A, Mecca G, Bertani T, Garattini S (1993). Prognosis of untreated patients with idiopathic membranous nephropathy. N Engl J Med.

[24] Ponticelli C, Zucchelli P, Passerini P, Cesana B, Locatelli F, Pasquali S (1995). A 10-year follow-up of a randomized study with methylprednisolone and chlorambucil in membranous nephropathy. Kidney Int.

[25] Jha V, Ganguli A, Saha TK, Kohli HS, Sud K, Gupta KL (2007). A randomized, controlled trial of steroids and cyclophosphamide in adults with nephrotic syndrome caused by idiopathic membranous nephropathy. J Am Soc Nephrol.

[26] Stasi R, Stipa E, Masi M, Cecconi M, Scimo MT, Oliva F (1995). Long-term observation of 208 adults with chronic idiopathic thrombocytopenic purpura. Am J Med.

[27] Neylon AJ, Saunders PW, Howard MR, Proctor SJ, Taylor PR, Northern Region Haematology G. Clinically significant newly presenting autoimmune thrombocytopenic purpura in adults: a prospective study of a population-based cohort of 245 patients. Br J Haematol. 2003;122:966–74. 10.1046/j.1365-2141.2003.04547.x 12956768

